# Neural Correlates of the Time Marker for the Perception of Event Timing

**DOI:** 10.1523/ENEURO.0144-16.2016

**Published:** 2016-09-21

**Authors:** Kaoru Amano, Liang Qi, Yoshikazu Terada, Shin’ya Nishida

**Affiliations:** 1 Center for Information and Neural Networks (CiNet), National Institute of Information and Communications Technology, Suita City 565-0871, Osaka, Japan; 2 Precursory Research for Embryonic Science and Technology (PRESTO), Japan Science and Technology Agency, Saitama 332-0012, Japan; 3 Graduate School of Frontier Sciences, The University of Tokyo, Kashiwa 277-8561, Chiba, Japan; 4 NTT Communication Science Laboratories, Nippon Telegraph and Telephone Corporation, Atsugi 243-0198, Kanagawa, Japan

**Keywords:** MEG, motion, synchrony, time marker, timing perception

## Abstract

While sensory processing latency, inferred from the manual reaction time (RT), is substantially affected by diverse stimulus parameters, subjective temporal judgments are relatively accurate. The neural mechanisms underlying this timing perception remain obscure. Here, we measured human neural activity by magnetoencephalography while participants performed a simultaneity judgment task between the onset of random-dot coherent motion and a beep. In a separate session, participants performed an RT task for the same stimuli. We analyzed the relationship between neural activity evoked by motion onset and point of subjective simultaneity (PSS) or RT. The effect of motion coherence was smaller for PSS than RT, but changes in RT and PSS could both be predicted by the time at which an integrated sensory response crossed a threshold. The task differences could be ascribed to the lower threshold for PSS than for RT. In agreement with the psychophysical threshold difference, the participants reported longer delays in their motor response from the subjective motion onset for weaker stimuli. However, they could not judge the timing of stimuli weaker than the detection threshold. A possible interpretation of the present findings is that the brain assigns the time marker for timing perception prior to stimulus detection, but the time marker is available only after stimulus detection.

## Visual Abstract

**Figure:**
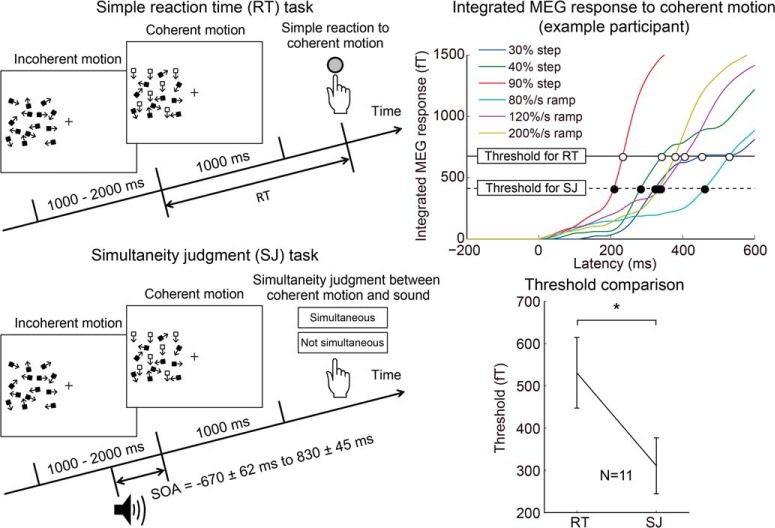

## Significance Statement

While reaction time (RT) is substantially affected by diverse stimulus parameters, subjective temporal judgments quantified by the point of subjective simultaneity (PSS) are relatively accurate. We found that both RT and PSS could be explained by the threshold detection mechanisms of the integrated sensory signals. The difference between RT and PSS was ascribed to the lower threshold for PSS than RT.

## Introduction

Accurate estimation of the time course of external events is a fundamental ability of animals for proper interaction with the dynamic environment. Human perception of event timing can be behaviorally assessed by asking participants to judge which of two stimuli came first [temporal order judgment (TOJ)] or whether they were simultaneous or not [simultaneity judgment (SJ)]. Generally, there is a disagreement between the subjective event timing, indicated by the point of subjective simultaneity (PSS) between the two stimuli, and the processing time required for stimulus detection inferred from the latency of a motor response to a stimulus onset, known as the simple reaction time (RT). Specifically, although RT is substantially affected by stimulus amplitude, type, and modality, PSS, estimated from subjective temporal judgments, is affected less by, and is thus more stable against, stimulus variations (Jaśkowski, 1992, 1993, 1996; Tappe et al., 1994; Cardoso-Leite and Gorea, 2010).

What are the neural mechanisms underlying this discrepancy? Whereas the variation of RT can be quantitatively explained by a model wherein a decision is made when the sensory evidence, accumulated over time by a leaky integrator, exceeds a threshold (Maunsell and Cook, 2002; Mazurek et al., 2003; Smith and Ratcliff, 2004; Amano et al., 2006; Ratcliff and McKoon, 2008), the neural mechanism underlying subjective event timing remains obscure. To account for the discrepancy between PSS and RT, some researchers argue that different sensory pathways, presumably ventral and dorsal visual pathways, are responsible for PSS and RT, respectively (Tappe et al., 1994; Steglich and Neumann, 2000). Other researchers argue that both RT and PSS reflect the neural response of the same pathway, but are determined in different ways. In other words, PSS is determined by an event “time marker” different from the physical timing of the event detection. A candidate time marker is the peak of the stimulus-evoked response (Sternberg and Knoll, 1973). Alternatively, the time marker may be determined by an evidence accumulation mechanism similar to that determining RT, but the criterion is set lower for PSS than for RT (Miller and Schwarz, 2006), or vice versa (Cardoso-Leite et al., 2007). The debate about the mechanisms underlying the perception of event timing has been primarily based on psychophysical data, and quantitative assessments based on neural data remain to be reported.

The present study examined the relationship between neural activity measured by magnetoencephalography (MEG), RTs, and PSS measured by SJ, for the same set of coherent motion stimuli. Although previous studies showing the dissociation of PSS and RTs primarily used TOJ (Jaśkowski, 1992, 1993,1996; Tappe et al., 1994; Cardoso-Leite and Gorea, 2010), here we used SJ since recent studies suggest that SJ is more stable than TOJ for the estimation of PSS (van Eijk et al., 2008; Fujisaki and Nishida, 2009). To significantly change the PSS, as well as RT, we manipulated the temporal profile of motion coherence presentations. Our results show that not only RT, but also PSS, could be predicted by the timing when the integrated sensory response crossed a threshold, with the threshold being lower for PSS than for RT. This finding, together with the results of subsidiary experiments, indicates that the time marker for the perception of event timing is assigned near the stimulus onset retrospectively, after stimulus detection.

## Materials and Methods

In the first experiment, MEG responses during the SJ task between a coherent motion onset and a beep were measured. RTs to the motion stimuli used for the SJ task were measured in a separate session. In the second experiment, RTs as well as the subjective delay in the motor response relative to the coherent motion onset were measured. PSS was also measured with the SJ task using the same motion stimuli in a separate session. In the third experiment, we measured the psychophysical threshold for motion coherence in the motion direction judgment task, as well as for the SJ task. The second and third experiments were conducted without MEG recording.

### General methods

#### Participants

Eleven participants (all males) participated in the first experiment, while 11 (7 males and 4 females) and 3 (all males) participants who were different from those in the first experiment participated in the second and third experiments, respectively. All participants provided written informed consent. All experiments were approved by the Safety Committee and the Ethics Committee of the University of Tokyo and the National Institute of Communication and Technology. The experiments were conducted in accordance with the Declaration of Helsinki.

#### Stimulus presentation

Stimuli were generated using a ViSaGe graphics system (Cambridge Research Systems). In the first experiment, the stimuli were presented by a digital light processing projector (V-1100Z, PLUS) onto a translucent screen. In the second and third experiments, the stimuli were presented on a cathode ray tube display (P1130, DELL). For all of the experiments, the refresh rate was 60 Hz, and the pixel resolution was 800 × 600 (40° × 30°).

We used random-dot coherent motion onset (transition from incoherent to coherent motion), which selectively activates the human MT complex (hMT+) and higher areas (Nakamura et al., 2003; Amano et al., 2006, 2012). On the dark background, white random dots (each subtending 0.16° × 0.16°, dot density, 8%) were presented within a square region in the left hemifield. The center of the right edge of the 10° × 10° stimulus area was 5° left of a fixation cross centered on the screen. A stimulus sequence consisted of incoherent motion followed by coherent motion. During the incoherent motion period, all dots moved randomly in one of eight directions. During the coherent motion period, a given proportion of the total number of dots moved in the same direction, whereas the remaining dots moved randomly in one of the seven remaining directions. The direction of the coherent motion was randomly chosen between upward and downward directions with equal probability to avoid adaptation to a specific direction. The speed of each dot was 8°/s (8 min jump for every 60 Hz frame). The duration of each dot was 33 ms (two frames).

In the SJ task, the sound was presented to the participants via an air-tube earphone (first experiment) or a pair of headphones (second and third experiments).

### First experiment

#### Procedure

In the first experiment, we used two types of temporal changes for the motion coherence: step and ramp stimuli. In the step stimulus, the coherence of the random dot motion abruptly changed from 0% to 30%, 40%, or 90%. In the ramp stimulus, the coherence gradually changed at the rate of 80, 120, or 200%/s from 0%. The reason for using two types of temporal changes is that the results from our preliminary experiment showed that the step and ramp stimuli affect the simultaneous perception differently. While a coherence change of the step stimulus had a minor effect on PSS, a slope change of the ramp stimulus had a major effect. To select the proper model of the underlying process, as described below, it was critical to produce significant stimulus-dependent variations in subjective simultaneity. In both conditions, incoherent motion was presented for a duration randomly varied between 1000 and 2000 ms, which was followed by coherent motion for 1000 ms. For the ramp stimuli at the rate of 120 and 200%/s, after the coherence reached 100%, it was kept at 100% until the coherent motion period (1000 ms) was over. The onset timing of ramp stimuli was defined as the timing when motion coherence started to increase from 0%. For both the step and ramp stimuli, after the coherent motion period ended, all dots disappeared.

**Figure 1.**
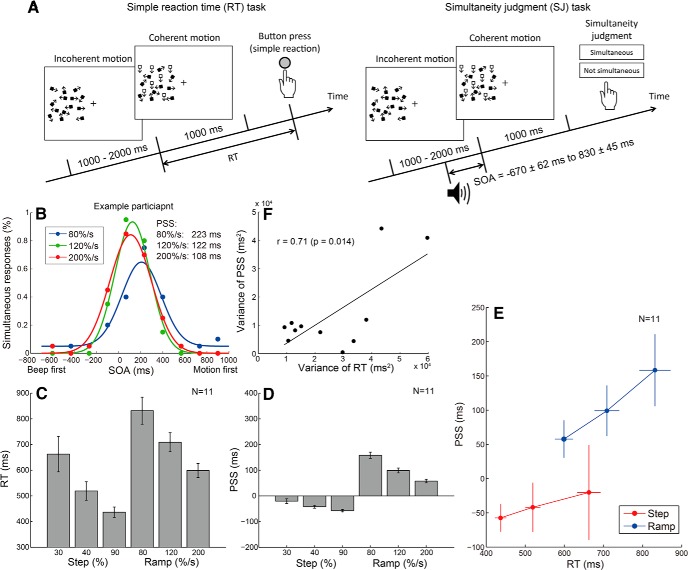
Dissociation between two measures of timing perception. ***A***, Stimulus configuration of the simple RT and SJ tasks. In both tasks, we used two types of temporal changes of the motion coherence: step (30%, 40%, and 90%) and ramp (80, 120, and 200%/s; for details, see Materials and Methods). ***B***, Example data from the simultaneity judgment task with three levels of ramp stimuli. The horizontal axis shows the SOA between the coherent motion onset and a beep, while the vertical axis shows the percentage of simultaneous responses. The PSS was defined as the weighted average of SOA values based on the percentage of simultaneous responses. ***C***, Averaged RTs for step and ramp stimuli. Error bars indicate SEs across participants. ***D***, Averaged PSS for step and ramp stimuli. Error bars indicate SEs across participants. Both RT and PSS decrease with increasing stimulus amplitude, but the decrease is much larger for RT than for PSS. ***E***, Comparison between the RT and PSS for both the step and ramp stimuli. Error bars indicate SEs across participants. The steeper slope for the ramp stimuli suggests that the amplitude of ramp stimuli has a larger effect on the PSS than the step stimuli. Please note that *x*- and *y*-axes are scaled differently. ***F***, Correlation between the variance of RT across stimuli and that of PSS. Each dot represents the data of individual participants. A significant correlation supports the result that the same integrated signal could account for both RT and PSS variations (Fig. 3).

Participants performed one of the following two tasks for these stimuli: a simple RT task and an SJ task (Fig. 1*A*). In the simple RT task, participants manually responded to the onset of coherent motion. In this experiment, a 0% coherent motion condition (no stimulus change) was included in addition to the step and ramp coherent motion stimuli. Coherent motion onsets at each coherence level and a 0% coherent motion onset were presented 32 times in random order. Participants were asked to press a key as soon as they detected coherent motion, but not to react if they did not perceive coherent motion. The time from coherent motion onset to the timing of the motor response was defined as the RT. With this task, we measured the objective time necessary for event detection.

In the SJ task, the coherent motion onset was presented with a beep (1800 Hz, 10 ms), and participants judged whether motion and sound were simultaneous or not by pressing one of two buttons. In each trial, the auditory stimulus was provided with a different stimulus-onset asynchrony (SOA; the timing difference between the coherent motion onset and the beep; positive values indicate the motion onset followed by the beep). The SOA of each trial was randomly selected from 10 levels that were determined for each participant in a preliminary experiment. On average, SOAs ranged from −670 ± 62 to 830 ± 45 ms. For all motion conditions, the stimulus at each SOA level was presented 20 times, resulting in 200 trials for each motion condition. We determined the percentage of the simultaneous responses as a function of the SOA, and defined the weighted average of SOA values based on the percentage of the simultaneous responses as the PSS. Because we repeatedly calculated the PSS in a bootstrap (BS) procedure with 10,000 replications for each motion condition to evaluate the reliability of the PSS estimation, we used the weighted average because it was much faster to compute than fitting a Gaussian function to the SJ responses. We, however, confirmed that the PSS estimated by the weighted average was very similar to that estimated by fitting a Gaussian function (*r* = 0.99).

#### MEG measurement

MEG responses during the SJ task were recorded using a whole-head MEG system (PQ2440R, Yokogawa) in a magnetically shielded room. The color of the fixation cross changed from white to red at coherent motion offset. Participants were instructed to respond after the fixation color change to reduce contamination by the motor component of the response. Data were sampled at 500 Hz with a 200 Hz low-pass filter and a 0.3 Hz high-pass filter. Our custom-made MEG system has 230 axial gradiometers (∂Bz/∂z) and 70 vector sensors, each consisting of one axial gradiometer (∂Bz/∂z) and two planar gradiometers (∂Bx/∂z, ∂By/∂z). In the current study, 300 axial gradiometers were used for the analysis, which made the comparison with previous studies easier. For each motion condition, we averaged MEG responses over 200 trials for 10 different SOA conditions. We did not separately analyze the responses for different SOAs, since we were interested in the neural response to visual stimuli that were common to different SOA conditions. Trials containing eye blinks, eye movements, muscle artifacts, or signal jumps were rejected off-line from further analysis.

#### Models of detection timing and time marker

To find the model that could best account for the changes in RT and PSS using the MEG response, we first extracted the time course of visual response amplitude from the averaged MEG responses for each stimulus condition. Then, we compared three models based on different aspects of the time course—the peak detector model, level detector model, and integrator model (Figure 3, A, B)—while considering the difference in the number of parameters and noise susceptibility across models.

The models used the time course of the visual response amplitude extracted by signal space projection (SSP) analysis (Tesche et al., 1995). To reduce contamination of the auditory response, we used not only the peak MEG response evoked by the strongest visual stimulus (90% step) but also the largest peak MEG response averaged with respect to the timing of auditory stimuli (either M100 or M200), as the spatial patterns for the SSP analysis. The root mean square of the 300 gradiometers was used to define the peak latency. We then obtained the time courses of visual and auditory response amplitudes by calculating the weight of the normalized peak spatial patterns that best accounted for the spatial pattern at each latency. Because coherent motion onset selectively activates the hMT+ and higher areas, and the peak visual response is known to originate from the hMT+ (Nakamura et al., 2003; Amano et al., 2006, 2012), the time course of the visual response most likely represents the hMT+ response amplitude. Before the SSP analysis, the averaged response over trials with respect to each stimulus onset was baseline (−200 to 0 ms) corrected and bandpass filtered at 1–40 Hz. The absolute values of the time course of the weights of visual responses were inputs to the models (because negative weights represent electric currents in the opposite direction to positive weights, not a decrease in response).

After extraction of the time course using a bandpass filter and the SSP analysis, a model-based analysis was conducted. For each participant, the PSS and median RT of each visual stimulus were compared with the latency predicted from models applied to the time course of the visual response amplitude. The peak detector model assumed that a stimulus is detected or the time marker is assigned when the visual response reaches its peak amplitude. The level detector model assumed that a stimulus is detected or the time marker is assigned when the visual response exceeds a given threshold. The integrator model assumed that a stimulus is detected or the time marker is assigned when the integrated visual response exceeds a given threshold. In the integrator model, MEG responses were convolved with a low-pass exponential filter, $exp\left(\frac{t}{\tau }\right)$. We used τ values of 100, 500, and 1000 ms, and ∞ (τ = ∞ corresponds to the full integrator without leakiness). The integrator models integrated response values only when the response exceeded the average plus 1 SD of the response during the baseline period (i.e., from −200 to 0 ms relative to the coherent motion onset; Amano et al., 2006).

For RTs, we assumed that a motor response follows stimulus detection with a postdetection delay that is unaffected by motion stimulus amplitude. For PSS, we assumed that the time marker for the beep is unaffected by motion stimulus amplitude. Therefore, if a model successfully predicted RT or PSS, the slope of RT versus detection latency function or the slope of PSS versus the time-marker latency function should be 1. To quantitatively compare model performance, a unit slope line was fitted to the scatter plot between RT/PSS (*x*-axis) and the latency predicted by each model (*y*-axis). The mean squared error (MSE) from the best-fitted unit slope line, which is the residual sum of squares averaged across stimulus conditions, was then calculated. This MSE represents how the detection timing or the time-marker variations are in accordance with the RT or PSS variations, respectively. The MSE accounts not only for any poorly correlated data with a slope of 1, but also for any highly correlated data with a slope higher or lower than 1. As we describe below, MSE is a biased estimator of prediction error, so we corrected for the bias.

For the latter two models, we determined the threshold value for each participant in the following way. At each threshold level, a line of unit slope was fitted to the scatter plot between RT/PSS as the *x*-axis and the latency predicted by the model (detection latency or time-marker latency) as the *y*-axis, and the MSE from the best-fitted line of unit slope was calculated. We then searched for the threshold that minimized the MSE. The stimulus conditions for which the time course of the visual response amplitude extracted by the SSP analysis did not exceed the noise level (2 SDs during the baseline period) were excluded from the analysis. The mean (±SE) number of excluded conditions was 0.55 ± 0.06.

#### Model comparison

The number of parameters differs across models (the level and integrator models have a threshold parameter that is not present in the peak detector model). In addition, noise susceptibility might differ across models. Therefore, for the statistical comparisons across models, we took into account both the difference in the number of parameters and the susceptibility to noise. The aim of our analysis was to find the best model in the sense of the following prediction error (PE):

$$PE(k)\;≔\;\underset{\theta_{k}}{min}\frac{1}{n}\overset{n}{\underset{i=1}{\sum }}[E[Y|s_{i}]-g_{k}(E[X|s_{i}]|\theta_{k}){]}^{2},$$

where *n* is the number of stimulus conditions (normally 6, but the number can be smaller, see above for details), $E[Y|s_{i}]$ is the expected value of RT/PSS for the stimulus amplitude $s_{i}$, $g_{k}$ is the *k*th model, $E[X|s_{i}]$ is the expected value of the MEG response time course (extracted by the SSP method) for the stimulus amplitude $s_{i}$, and $\theta_{k}$ is the parameter vector of the *k*th model. Although MSE is often used as the estimator of PE(k), MSE is a biased estimator of PE(k). Typically, increases in the number of parameters can result in the model overfitting the data. To correct for bias in MSE, we employed a bootstrap method, as with the extended information criterion (EIC) (Ishiguro et al., 1997). Henceforth, we refer to this bias-corrected estimator of PE(k) as bias-corrected MSE.

Here we note that usual regression models, assumed in Akaike information criterion (AIC) and Bayesian information criterion (BIC), are not appropriate as a generative model of our data. The regression models for our data can be represented by the following:

$$Y_{i}=g_{k}\left(X_{i}\right)+\epsilon_{i}\;(i=1,\ldots ,n),$$

where *i* is an index of stimulus amplitude $s_{i}$, and $\epsilon_{i}$ represents independent and identically distributed (i.i.d.) random variables with $E\left[\epsilon_{i}\right]=0$ and $Var\left[\epsilon_{i}\right]=\sigma^{2}<\infty$. In the usual regression model, $X_{i}$ is not random for a fixed stimulus amplitude, $s_{i}$. In our models, however, not only $Y_{i}$ (RT/PSS) but also $X_{i}$ (MEG response time course) for a given stimulus amplitude, $s_{i}$, is random. Therefore, our models do not fit the scheme of usual regression models. Moreover, even if we enforced the usual regression model for our data, the sample size of six is too small to evaluate the candidate models correctly since AIC and BIC are based on asymptotic results. Therefore, we needed to use a bootstrap method to correct for bias in MSE, as is used for the extended information criterion (Ishiguro et al., 1997).

Below, we describe how to calculate the bias-corrected MSE for the model. Each MEG response time course is assumed to be i.i.d. The first-level BS samples $D_{i}^{*}\;(i=1,\;\ldots ,B_{1})$ were generated by resampling trials with replacement. For each first-level BS sample, $D_{i}^{*}$, we computed the optimal parameter $\hat{\theta }{(D}_{i}^{*})$ and calculated the MSE, $MSE(D_{i}^{*})$. Here, we note that for the peak detector model, the *y*-intercept in the plot between peak latency and RT/PSS data was the only parameter (the slope was fixed at 1). The *y*-intercept for RT corresponds to the motor delay, while that for PSS corresponds to the timing of the time marker for the auditory response. For the level and integrator models, the parameters were the *y*-intercept and threshold. For each first-level BS sample, $D_{i}^{*}$, we generated the second-level BS samples, $D_{ij}^{†}(j=1,\ldots ,B_{2})$, and then for each second-level BS sample, we calculated the mean squared error, $\;MSE(D_{ij}^{†}|\hat{\theta }(D_{i}^{*}))$, with the optimal parameter $\hat{\theta }(D_{i}^{*})$ of $D_{i}^{*}$. Then we defined $\overset{˜}{C}{(D}_{i}^{*})$ as follows:

C˜(Di*) : =MSE(Di*)-1B2∑j=1B2MSE(Dij†|θ^(Di*)).

$\overset{˜}{C}{(D}_{i}^{*})$ is the difference between the MSE for one bootstrap sample $D_{i}^{*}$, with its optimal parameters, and the MSE for other bootstrap samples $D_{ij}^{†}(j=1,\ldots ,B_{2})$, with the parameters optimal for $D_{i}^{*}$ (not necessarily optimal for $D_{ij}^{†}(j=1,\ldots ,B_{2})$ themselves). $\overset{˜}{C}{(D}_{i}^{*})$ represents an underestimation of the MSE value caused by overfitting and is negative in most cases. In case a model has generalization ability, $\overset{˜}{C}{(D}_{i}^{*})$ approaches 0. Conversely, if a model overfits the data, the absolute value of $\overset{˜}{C}{(D}_{i}^{*})$ gets larger.

By averaging $\overset{˜}{C}{(D}_{i}^{*})$ across the first-level BS samples, we obtained the estimator $\hat{C}$ of the bias term. The bias-corrected estimator of PE (i.e., the bias-corrected MSE) is obtained as follows:

MSE-C^ (C^ : =1B1∑i=1B1C˜(Di*)).

In our analysis, we set $B_{1}=100$ and $B_{2}=100.$

For our model selection based on the bias-corrected MSE, we applied the Shimodaira–Hasegawa (SH) test (Shimodaira, 1998), which verifies whether each model is significantly worse than the best model. Thus, we obtained a confidence set of models, which included the best model, with an error smaller than the fixed significance level (0.05). This test requires one more level of BS samples for estimating the variance of the bias estimator $\hat{C}$, but it is computationally difficult to use a third level of bootstrapping. Fortunately, since the variance of $\hat{C}$ was relatively small compared with the variance of MSE, the bias term was regarded as a fixed value. The SH test was conducted using 1000 BS samples on the bias-corrected MSE summed across participants.

### Second experiment

In the second experiment, the coherence of the random dot motion abruptly changed from 0% (1000–2000 ms) to 8%, 16%, or 32% (1000 ms). As in the first experiment, participants performed one of the following two tasks for these stimuli: simple RT and SJ tasks.

In the simple RT task, participants manually responded to the onset of coherent motion, after which they also reported the subjective delay of their motor response relative to the stimulus onset (Fig. 6*A*). Subjective delay was chosen from six levels. For creating an internal representation of subsecond time, a practice session was conducted before the experiment to report the subjective delay in RT. Coherent motion (100% coherence) with a duration of 100, 167, 250, 350, 467, or 600 ms was presented randomly, and participants selected the perceived duration from six levels. The correct answer for durations of 100–600 ms was 1 to 6, respectively. During the practice session, feedback of the correct answer was provided, and the practice session was conducted until the percentage correct exceeded 70%, which normally took 10–20 min. After the practice, coherent motion onsets at each coherence level as well as a 0% coherent motion onset were presented 100 times in random order, and RT, as well as its subjective delay, were measured. The procedure for the SJ task was the same as in the first experiment.

### Third experiment

In the third experiment, which measured psychophysical thresholds, the coherence of the random dot motion abruptly changed from 0% (1000–1500 ms) to 1%, 2%, 4%, 8%, 16%, or 32% (1000 ms). Participants performed both a motion direction judgment and SJ in each trial (Fig. 7*A*). After participants chose one of the two directions of coherent motion (upward vs downward), they also reported whether the coherent motion onset was simultaneous with a beep. SOA between the coherent motion onset and a beep was −500 or 500 ms (not simultaneous), or 0 ms (simultaneous). A stimulus with each SOA was presented 52 times for each coherence level (1%, 2%, 4%, 8%, 16%, or 32%), so that asynchronous and synchronous stimuli were presented at the same frequency.

## Results

### Neural correlates of the time marker (first experiment)

Participants performed the RT and SJ tasks for the same random-dot coherent motion stimuli (Fig. 1*A*). Participants responded to the coherent motion onset as soon as possible (RT task) or judged simultaneity between a coherent motion onset and a beep presented at various times relative to the coherent motion onset (SJ task). For both tasks, motion coherence increased from 0% to 30%, 40%, or 90% abruptly (step stimuli), or at a rate of 80, 120, or 200%/s gradually (ramp stimuli). For the SJ task, PSS was computed from the percentage of simultaneous responses plotted as a function of SOA for each condition (Fig. 1*B*, example data for ramp stimuli).

The stimulus miss ratio in the RT task was 10% for all stimuli, except for the 30% step stimulus (41% miss ratio). Figure 1, *C* and *D*, shows the median RT and PSS, respectively, averaged across all participants. Both the RT and PSS decreased with increasing stimulus amplitude, but the change was much larger for RTs than for the PSS. RTs changed by >200 ms for both the step and ramp stimuli. In contrast, the change in PSS was <50 ms for the step stimuli, while for the ramp stimuli it was ∼100 ms. Figure 1*E* compares the RTs and PSS for both the step and ramp stimuli. Significantly steeper slopes for the ramp stimuli than for the step stimuli (*p* = 0.042, Wilcoxon signed rank test) suggest that the amplitude of the ramp stimuli had a larger effect on the PSS than the step stimuli, while the amplitude of the step and ramp stimuli had a similar effect on the RT. The dissociation between the RT and PSS (or between the objective time necessary for event detection and the subjective event timing) is consistent with previous studies that used TOJ as the psychophysical task for PSS estimation (Jaśkowski, 1992, 1993, 1996; Tappe et al., 1994; Cardoso-Leite and Gorea, 2010).

**Figure 2.**
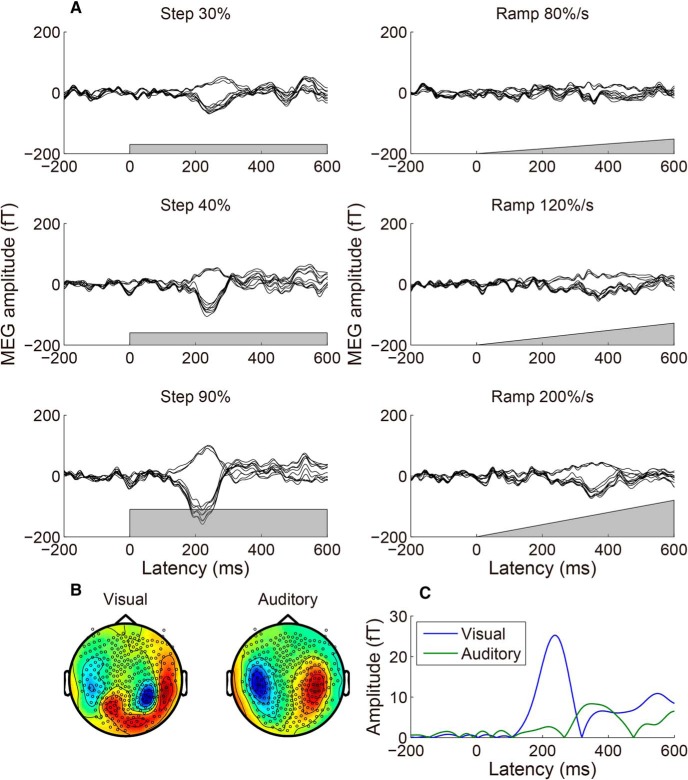
An example of MEG responses to step and ramp motion onsets and the extracted visual response time course. ***A***, MEG response time courses averaged across trials for a typical participant. Data from 10 sensors that showed the greatest response for the 90% step stimulus at the peak latency are overlaid, and the gray region in each panel indicates the motion coherence time course. For the step stimuli, the MEG responses peaked at ∼230 ms, and the amplitude increased with motion coherence. For the ramp stimuli, the response peak was less clear and the peak latency was much longer. ***B***, Normalized spatial pattern (topographic map) of the visual and auditory responses used in the SSP analysis, for the participant shown in ***A***. The left image shows the peak MEG response evoked by the strongest visual stimulus (90% step), while the right image shows the largest peak MEG response averaged with respect to the timing of auditory stimuli. The auditory peak was either M100 or M200 (it was M100 for eight participants, and was M200 for three participants, including the participant shown here). ***C***, The time course of visual responses for the 90% step stimulus, extracted by the SSP method (Tesche et al., 1995). The spatial pattern at each latency was decomposed into visual and auditory responses by calculating the best weight of the spatial pattern of visual and auditory responses (***B***), and the absolute value of the weight was defined as the response amplitude.

Figure 2*A* shows typical overlaid MEG response time courses evoked by the coherent motion onsets, measured during the SJ task. The response was averaged across 200 trials for one participant. The gray region indicates the time course of motion coherence. The responses to a beep canceled out because the beep was presented randomly at a variety of SOAs with respect to the coherent motion onsets (see below for an additional analysis). For the step stimuli, the MEG responses peaked at ∼230 ms, and the amplitude increased with the motion coherence. For the ramp stimuli, the response peak was less clear, and the peak latency was much longer. We then extracted the time course of the weight of the normalized peak spatial pattern, most likely originating from hMT+ (Nakamura et al., 2003; Amano et al., 2006, 2012), by SSP (Tesche et al., 1995). To reduce contamination by the auditory response, we used not only the peak MEG response evoked by the strongest visual stimulus (90% step), but also the peak MEG response averaged with respect to the timing of auditory stimuli (either M100 or M200, the time course is not shown; for details, see Materials and Methods), as the spatial patterns for the SSP analysis. Figure 2, *B* and *C*, shows the visual and auditory spatial patterns used for the SSP analysis (the peak latency was 233 and 181 ms (M200), respectively), and the extracted time course for the 90% step for the participant shown in Figure 2*A*. The mean (±SE) peak latency of the visual responses was 228 ± 10 ms, which is consistent with previous studies that used a similar visual stimulus (Nakamura et al., 2003; Aspell et al., 2005). The averaged latency of the M100 and M200 peaks of the auditory response were 104 ± 2 and 204 ± 9 ms, respectively, which is again consistent with previous literature (Roberts et al., 2000). We used the spatial pattern of the larger peak (M100 for eight participants and M200 for three participants), because the purpose of the SSP analysis is to extract contamination of an auditory response. The averaged latency of the auditory response used was 125 ± 12 ms.

Using the weight time course of the visual response amplitude, we sought the best model that could account for the RT and PSS variations. The latency predicted by the model corresponds to the timing of stimulus detection (for RT) or the timing of the time marker (for PSS). The peak detector model assumed that the stimulus is detected or the time marker is assigned when the visual response reaches a peak. The level detector and integrator models assume that the stimulus is detected or the time marker is assigned when the visual response or the integrated visual response crosses a certain threshold. The only difference between the level detector and integrator models is the existence of temporal integration. The threshold was optimized for the level detector and integrator models.

**Figure 3.**
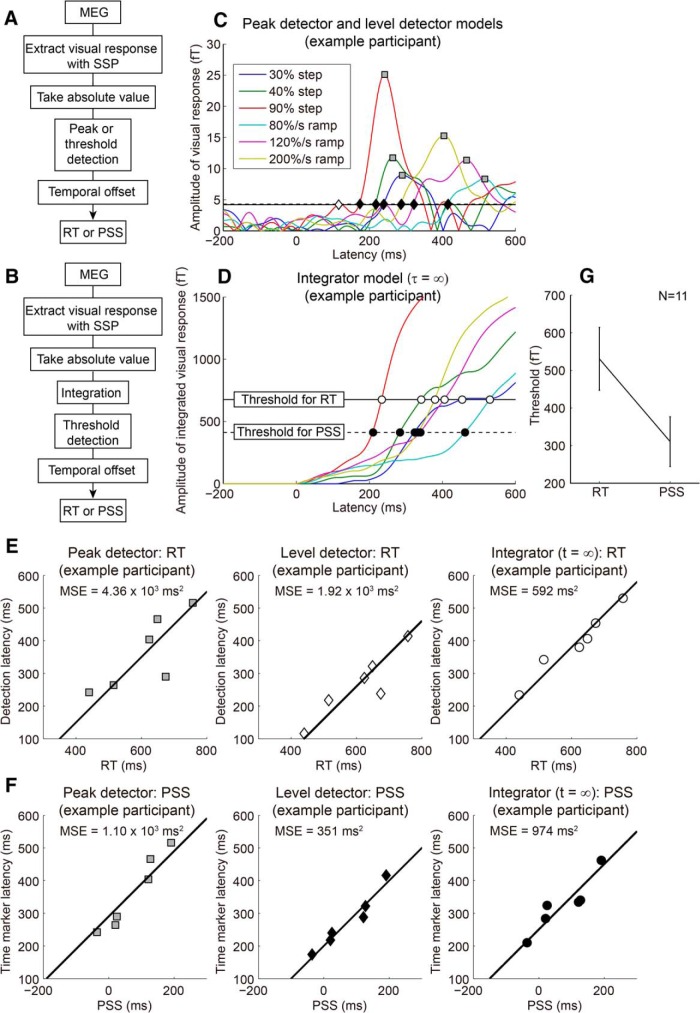
Neural correlates of RT and PSS. ***A***, ***B***, Schematic of the peak/level detector models (***A***) and the integrator model (***B***). ***C***, The time course of visual responses for a typical participant. Solid and dotted lines represent the optimized threshold of the level detector model for RT and PSS, respectively (they were almost the same for this participant). Gray squares represent the latencies predicted by the peak detector model (common across the detection latencies for RT and time-marker latencies for PSS). Diamonds represent the latencies predicted by the level detector model (open and filled diamonds represent detection latencies for RT and time-marker latencies for PSS, respectively). ***D***, The time course of integrated visual responses (τ = ∞) for the same participant as shown in ***A***. Solid and dotted lines represent the optimized threshold of the integrator model for RT and PSS, respectively. Open and filled circles represent the detection latencies and time-marker latencies, respectively. The threshold for PSS was lower than that for RT. ***E***, ***F***, Relationship between RT and detection latency (***E***), or between PSS and time-marker latency (***F***), for the participant shown in ***A*** and ***B***. Since we are interested in the model that quantitatively accounts for the RT or PSS variations, the model performance was evaluated by the MSE from the best-fitted line of unit slope, which takes into account not only poorly correlated data with a slope of 1 but also highly correlated data with a slope higher or lower than 1. ***G***, Comparison of the threshold for the integrator model (τ = ∞) that best accounts for RT and PSS variations. Error bars indicate SEs across participants. The threshold was significantly lower for PSS than for RT, suggesting that the stimulus content information, which is necessary for manual response, was not available at the timing of the time marker, and the event timing is postdictively perceived (Nishida and Johnston, 2002).

Figure 3*C* shows a typical example of the visual response amplitude with the latencies predicted by the peak detector (square) and level detector (diamond) models (Fig. 3*A*). It should be noted that the responses were measured only during the SJ task and that the RT task was performed outside the MEG. Figure 3*D* shows the integrated visual response amplitude (τ = ∞) with the latencies predicted by the integrator model (Fig. 3*B*). In the case of the integrator model, for example, coherent motion is detected when the integrated response crosses the RT threshold (Fig. 3*D*, open circles), while the time marker for the perception of event timing is assigned when the integrated response crosses the PSS threshold (Fig. 3*D*, filled circles). Figure 3E shows the relationship between RT and the latency predicted by the peak detector, level detector, and integrator models. Figure 3*F* shows the relationship between PSS and the latency predicted by each model. The model performance was evaluated as the MSE from the unit–slope line of best fit (Fig. 3*E*,*F*, lines). For the participant illustrated, the model performance for RT was best for the integrator model, while the performance for PSS was comparable across models.

**Figure 4.**
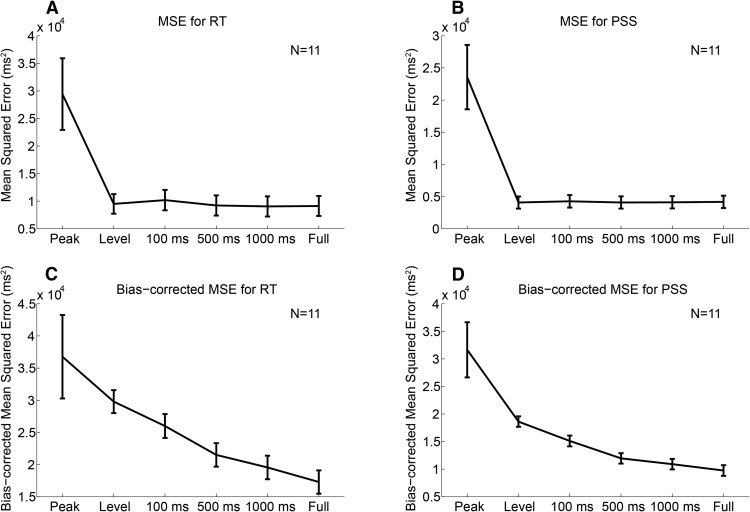
Comparison of models that account for the changes in RT and PSS using the MEG response. ***A***, ***B***, The MSE from the best-fitted line of unit slope for the scatter plot between RT/PSS (*x*-axis) and the latency predicted by the model (*y*-axis), as shown in Figure 3, *E* and *F*. The MSE was summed across participants, and the error bars indicate SDs across 1000 bootstrap samples. ***C***, ***D***, While MSE is a biased estimator of the prediction error, bias-corrected MSE accounts for the difference in both susceptibility to noise and the number of parameters across models. The bias-corrected MSE was summed across participants, and the error bars indicate SDs across 1000 bootstrap samples. ***A*** and ***C***, and ***B*** and ***D*** show the results for the RT and PSS data, respectively. The SH test (Shimodaira, 1998) performed on the bias-corrected MSE showed that the peak and level detector models were significantly worse than the best model for both RT and PSS (Table 1).

Figure 4 shows the MSE and the bias-corrected MSE for the RT (Fig. 4*A*,*B*) and PSS data (Fig. 4*C*,*D*), summed across participants (for details, see Materials and Methods). The bias-corrected MSE takes into account both the difference in the number of parameters and the susceptibility to noise across models. The comparison of MSE (Fig. 4*A*,*B*) and bias-corrected MSE (Fig. 4*C*,*D*) suggests that the absolute value of the bias term $\hat{C}$ (the difference between MSE and bias-corrected MSE) tended to be larger for the level detector model and the leaky integrator models with smaller leaky parameters. While MSE was similar across the level detector and the leaky integrator models regardless of the leaky parameter, bias-corrected MSE differed across models. The results of the SH test for RT performed on the bias-corrected MSE, wherein the null hypothesis is that the corresponding model is not different from the best model, showed that a confidence set of models was given by the leaky integrator models, with τ = 100, 500, and 1000 ms, and the full integrator model (Table 1). The peak detector and level detector models were significantly worse than the best model (*p* = 0.001 and *p* = 0.007, respectively). The results replicate previous MEG study results (Amano et al., 2006). An SH test for PSS (Table 1) also showed that a confidence set of models was given by the leaky integrator models with τ = 100, 500, and 1000 ms, and the full integrator model; the peak and level detector models were significantly worse than the best model (*p* = 0 and *p* = 0.016, respectively). These results suggest that the leaky/full integrator model accounted not only for the RT variation, but also for the PSS variation. Different integration parameter values resulted in different response slopes: a larger leak parameter τ corresponds to a shallower ramping slope, as is evident from the comparison between Figure 3, *C* and *D* (τ = 0 ms and ∞, respectively), which can explain the task differences. However, that the bias-corrected MSE as a function of τ was qualitatively similar between RT and PSS suggests that the task differences cannot be ascribed to the different ramping slopes between RT and PSS.

**Table 1:** p Values for the SH test on the bias-corrected MSE summed across participants

	Peak	Level	Leaky (τ = 100 ms)	Leaky (τ = 500 ms)	Leaky (τ = 1000 ms)	Full (τ = ∞)
RT	0.001	0.007	**0.052**	**0.304**	**0.488**	**0.906**
PSS	0	0.016	**0.124**	**0.338**	**0.509**	**0.889**

The null hypothesis is that the corresponding model is not different from the best model, while the alternative hypothesis is that the corresponding model is worse than the best model. Bold type indicates the models within a confidence set of models.

Given that both RT and PSS were best explained by the integrator model, what resulted in the difference between them? Figure 3*D* shows that the time marker was assigned to a time point earlier than the stimulus detection timing. The group data (Fig. 3*G*) showed that the threshold was significantly lower for PSS than for RT (*t*_(10)_ = 3.12, *p* = 0.011). Stimulus amplitude affects the slope of the increase in the neural response, as is evident in Figure 3*D*; the lower the threshold, the smaller the effect of the slope on the latency. The lower threshold for PSS than for RT corresponds to the smaller variation in PSS (Fig. 1*E*). Although the full integrator model (τ = ∞) is unrealistic, the results of physiologically plausible leaky integrator models (τ = 100, 500, and 1000 ms) were very similar to those shown in Figure 3 and indicated that the threshold was significantly lower for PSS than for RT (*t*_(10)_ = 2.48, *p* = 0.033; *t*_(10)_ = 2.77, *p* = 0.020; and *t*_(10)_ = 2.99, *p* = 0.014, respectively).

If the same integrated signal could account for both RT and PSS, this might imply a correlation between RT and PSS across participants. Namely, for the participants whose RT variation across stimuli was relatively large, the variation in PSS might also be relatively large. As we expected, Figure 1*F* shows that the variance of RT was significantly correlated with that of PSS (*r* = 0.71, *p* = 0.014). Although this supports the idea that the same integrated signal can account for both PSS and RT, we may have to refrain from drawing a definite conclusion at this stage, since the correlation was mainly driven by the data of two participants who showed exceptionally large stimulus-dependent variations in both tasks.

**Figure 5.**
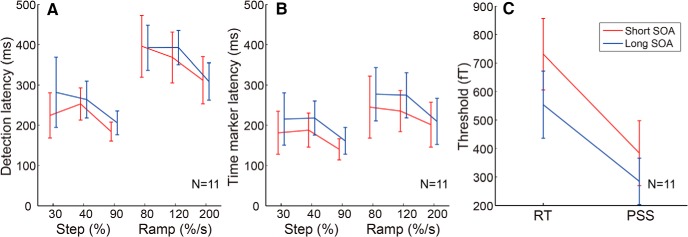
Effect of an auditory stimulus on the model latencies. ***A***, ***B***, Detection latency for RT (***A***) and time-marker latency for PSS (***B***) based on the full integrator model (τ = ∞), calculated separately for shorter and longer SOA trials. Similar latencies between shorter and longer SOA trials indicate that the auditory response was reasonably removed by the SSP analysis to extract the visual response time course, and had a negligible effect on the latencies predicted by the integrator model. ***C***, Comparison of the threshold for the integrator model (τ = ∞) that best accounts for RT and PSS variations, calculated separately for shorter and longer SOA trials. Error bars indicate SEs across participants. The threshold was significantly lower for PSS than for RT for both shorter and longer SOA trials (*p* = 0.03 and *p* = 0.001, respectively), replicating the analysis that used all of the trials (Fig. 3*G*).

In the current analysis, we used SSP (Tesche et al., 1995) to reduce the contribution of the auditory evoked response. To ensure that the model latencies (detection latency for RT and time-marker latency for PSS) were not affected by the auditory response, we separately averaged MEG responses across the trials with SOAs shorter than the median SOA and the trials with SOAs longer than the median SOA, and compared model latencies between them with the full integrator model (τ = ∞; Fig. 5*A*,*B*). 

Two-way ANOVAs (stimulus × SOA) showed that the main effect of SOA was not significant for both RT (*F*_(1,92.1)_ = 0.74, *p* = 0.392) and PSS (*F*_(1,92.0)_ = 2.19, *p* = 0.142), suggesting that model latency was similar for shorter and longer SOA trials. Additionally, the thresholds for the integrator model (τ = ∞), estimated separately for shorter and longer SOA trials, were significantly lower for PSS than for RT for both shorter and longer SOA trials (Fig. 5*C*; *p* = 0.03 and *p* = 0.001, respectively), replicating the analysis that used all trials (Fig. 3*G*). These analyses support the idea that the auditory evoked response does not affect our conclusions, although we cannot completely exclude the contribution of auditory responses.

From the results of the first experiment, we suggest that the brain assigns the time marker for motion onset to a time point earlier than the detection latency.

### Subjective response delay relative to subjective stimulus onset (second experiment)

The lower threshold for PSS than for RT implies that the difference between the time marker for the perception of event timing and the time point when the participant reaches a decision to initiate a motor response (Fig. 3*D*, the timing difference between open and filled circles on response time courses of the same color) is larger when the accumulated neural response develops slowly with a weak input compared with when it develops rapidly with a strong input. A delay in motor response initiation relative to subjective event timing might sound counterintuitive, given that the participants had to make a motor response as soon as they detected coherent motion. To see whether the event time course predicted from our MEG analysis is consistent with the subjective time course reported by the participants, a subsidiary psychophysical experiment was performed. Specifically, we tested whether the participants would perceive that the delay (from the subjective onset of the coherent motion) in their motor response was greater for weaker stimuli than for stronger stimuli, consistent with our model prediction.

**Figure 6.**
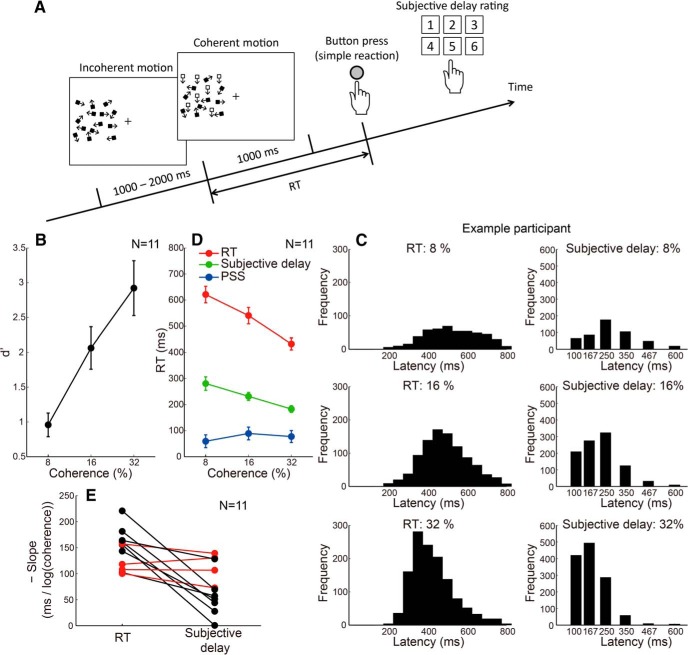
Subjective delay of motor response. ***A***, Stimulus configuration of the second experiment that measured the subjective delay of simple reactions. ***B***, Stimulus detectability, expressed in terms of *d*', as a function of motion coherence. Error bars indicate SEs across participants. ***C***, Representative histograms of RT and subjective delay of motor response for each motion coherence level. ***D***, The RT, subjective delay of RT, and PSS, as a function of motion coherence. Error bars indicate SEs across participants. ***E***, The slope of the fitted line for the plot between coherence level and RT/subjective delay for each individual participant. The subjective delay slope, on average, was shallower than the RT slope but was significantly <0 (*p* < 0.001). In addition, the RT and subjective delay slopes were similar for 4 of 11 participants (red lines).

In each trial of this experiment, participants performed the RT task by pressing a button as soon as they detected step onsets of 8%, 16%, and 32% coherent motion following an incoherent motion period (Fig. 6*A*). After the motor response, they had to report how much they felt their response was delayed relative to the subjective onset of the coherent motion. Specifically, they made a six-level rating of the subjective delay of their response. Before this experiment, a practice session was conducted to let the participant associate the numbers one to six with the coherent motion durations of 100, 167, 250, 350, 467, and 600 ms, respectively. The PSS in the SJ task was measured with the same set of coherent motion stimuli (8%, 16%, and 32%) in a separate session.

Since this experiment used relatively low motion coherences, participants missed the coherent motion onset in a significant number of trials. Figure 6*B* shows that the stimulus detectability, expressed in terms of *d*', increased as a function of motion coherence, and that the lowest coherence (8%) was close to the detection threshold (*d*' ≈ 1). Figure 6*C* shows representative histograms of RTs and subjective delay ratings. The delay rating numbers (1, 2, 3, 4, 5, or 6) correspond to the respective durations associated with the practice session (100, 167, 250, 350, 467, or 600 ms). The histogram tends to shift toward shorter latencies, with an increase in coherence not only for RT but also for the subjective response delay. Given the broader distribution of ratings for the 8% coherence, the longer subjective delay for weaker stimuli at least partly reflects the uncertainty of weak visual stimuli. Figure 6*D* shows RT, subjective response delay, and PSS as functions of motion coherence, averaged across participants. RT decreased by ∼200 ms as the coherence increased from 8% to 32%, while PSS was rather independent of coherence. The subjective response delay decreased with motion coherence (slope of the fitted line significantly <0, *P* < 0.001). The slope was similar between the subjective response delay and RT for 4 of 11 participants (Fig. 6*E*, red lines), indicating that they could accurately estimate to what extent their motor response was delayed relative to the subjective motion onset for weak versus strong stimuli. The similar slopes indicate that RT minus subjective delay is constant, which is consistent with a PSS that is independent of coherence. Although the slope was, on average, shallower for the subjective response delay than for RT (Fig. 6*E*), action is known to distort time estimation in various ways (Haggard et al., 2002; Hagura et al., 2012), and the validity of the associated quantity of the delay depends on how accurately each participant could perform this complex psychophysical task. That the participants could tell their responses were delayed more for weaker stimuli than for stronger stimuli implies that the participants had some awareness of their response delay relative to the subjective motion onset. This is in agreement with our hypothesis that the time marker is temporally close to the physical stimulus onset, which is earlier than the detection timing.

### Temporal judgment with subliminal stimulus onset (third experiment)

**Figure 7.**
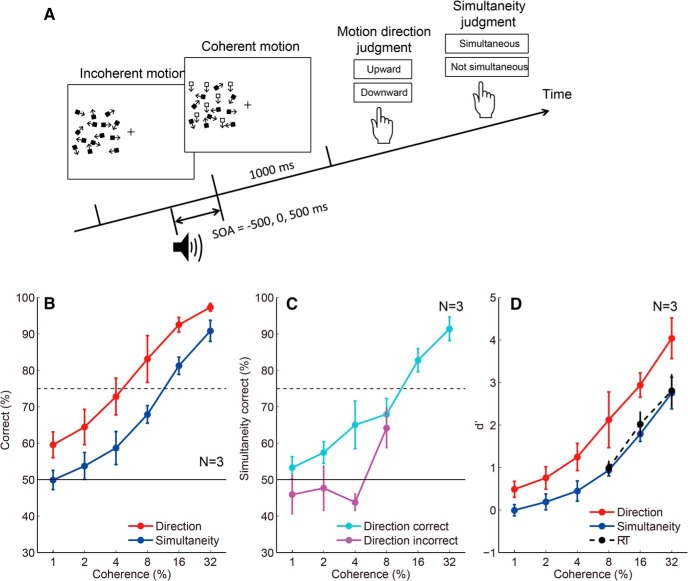
Comparison of the percentage correct between motion direction judgments and simultaneity judgments. ***A***, Stimulus configuration of the third experiment that measured the threshold for motion direction judgments and simultaneity judgments. ***B***, The percentage correct for motion direction judgments and simultaneity judgments. Error bars indicate SEs across participants. ***C***, The percentage correct for simultaneity judgments analyzed separately for the correct and incorrect trials for motion direction judgments. Error bars indicate SEs across participants. The percentage correct for simultaneity judgments for direction-incorrect trials is at the level of chance. ***D***, Comparison of *d*' for motion direction judgments, simultaneity judgments (third experiment), and a simple RT (second experiment) task. Error bars indicate SEs across participants. RT task *d*' values were lower than motion direction judgment task *d*' values; however, they were close to, but never lower than, simultaneity judgment task *d*' values.

Although the response threshold to determine the time marker for the perception of event timing is lower than that to initiate a motor response (Fig. 3*D*,*G*), the participants could not initiate a motor response until the accumulated neural evidence exceeded the lower threshold for the time marker. To understand the reason, we thought it necessary to clarify the relationship between the minimum stimulus amplitude needed to judge the stimulus onset timing (psychophysical threshold for timing), and the minimum stimulus amplitude needed to detect the stimulus (psychophysical threshold for detection). According to our model prediction, one might expect that the psychophysical threshold for timing is lower than that for detection, but this is not necessarily the case if we assume postdictive processing, as we discuss below. In this experiment, we had the participants make a motion direction judgment and an SJ for the same motion onset by manipulating motion coherence. In each trial, participants reported the direction of coherent motion (upward vs downward) as well as whether the coherent motion onset was simultaneous with a beep (Fig. 7*A*). Figure 7*B* shows that the percentage correct of motion direction judgment is systematically higher than that of SJ, with the psychophysical threshold (75% correct coherence), being ∼15% for simultaneity judgment and ∼5% for direction judgment. When we separately analyzed the correct and incorrect direction judgment trials, the SJ performance was at the chance level for the incorrect trials (Fig. 7*C*). The results indicate that the timing judgment was impossible when the stimulus amplitude was below the psychophysical threshold for detection.

One limitation of this experiment is that the psychophysical threshold for detection was estimated based on a binary direction discrimination task. On the other hand, in earlier experiments, RT was measured using a go/no-go task to the presence/absence of a coherent motion onset. To seek possible effects due to task differences, we compared the performance in the detection and simultaneity judgment tasks in the last experiment with the detection performance in the RT task in the second experiment, using *d*' as the common measure of sensitivity. The result (Fig. 7*D*) indicates that the *d*' value for the RT task was lower than that for the direction task, and was close to, but never lower than, the *d*' value for the simultaneity judgment task. Note that a significant number of false alarm responses (∼10%) to catch trials (0% coherence) for the RT task indicates that the participants did not set the response criterion excessively high to avoid false alarms. This result indicates that even though the time marker of a motion onset is assigned to a time point earlier than the detection timing the participants cannot access the motion onset timing for subliminal stimuli that do not eventually reach a psychophysical threshold for detection.

## Discussion

Stimulus amplitude affects RT and PSS differently (Fig. 1*C–E*), suggesting that they are based on different neural substrates. We found that both the RT and PSS were explained by the integrator model assuming the threshold detection mechanism of the integrated sensory signals (Fig. 3). The threshold for the integrator was found to be lower for PSS than for RT (Fig. 3*G*), which is consistent with the idea that the time marker for the perception of event timing is located prior to stimulus detection. Consistent with this result, we also found that participants could correctly report that the delay in motor response from the stimulus onset was longer for weaker stimuli than for stronger stimuli (Fig. 6). The final experiment indicated that the timing judgment was impossible for subliminal stimuli (Fig. 7).

Several models concerning the dissociation of perceptual and motor latencies have been suggested (Cardoso-Leite and Gorea, 2010). For a single pathway using a two-decision model, the peak latency model was one of the candidates to account for PSS (Sternberg and Knoll, 1973), but here showed worse performance than the integrator model (Fig. 4*D*, Table 1). Our results are consistent with the idea of Miller and Schwarz (2006) that a lower criterion for the subjective timing judgment (TOJ in their case) than for the RT task might be optimal. That is, the threshold for the TOJ task is optimal when it maximizes the probability of a correct order judgment, while the threshold for RT is optimal when it ensures a small number of false alarms. Therefore, the optimal threshold is lower for TOJ than for RTs. Cardoso-Leite et al. (2007) found that the variability of PSS was larger than that of RT, and interpreted this result to suggest that the threshold was lower for RT than for PSS, which seems to be inconsistent with the current result that the threshold for the integrated response was lower for PSS than for RT. We suggest that the variance of PSS reflects not only the sum of the variance of the sensory signals to be compared but also the noise of the timing comparator of sensory signals. Therefore, the larger variability of PSS does not necessarily indicate a lower threshold for motor latency (RT) than for perceptual latency (PSS).

The two independent pathway models suggest that RT and SJ are under the control of the dorsal and ventral pathways, respectively (Tappe et al., 1994; Steglich and Neumann, 2000). Although the current study did not directly test the single versus multiple pathway hypotheses, our result that a common integrated sensory response could explain both the RT and PSS is in accordance with a single-pathway model rather than a multiple-pathway model. The significant correlation between the variance of RT and that of PSS (Fig. 1*D*), and the correlation between RT and TOJ measured in each trial (Cardoso-Leite et al., 2007) are also consistent with the single-pathway model. Still, we cannot fully exclude the possibility that two independent pathways use exactly the same integrated signals. If we assume the same integrated signals, the lower threshold in the SJ task than in the RT task can be at least partly ascribed to the difference in stimulus certainty, because in the negative SOA conditions of the SJ task, an auditory stimulus can be a cue that the visual stimulus will appear soon. However, we believe that the difference in the threshold between the SJ and RT tasks cannot fully be ascribed to the difference in stimulus certainty, because even in the second experiment, in which no auditory stimulus was presented, we found an increase in the subjective delay of motor response for weaker stimuli.

According to the results of the two subsidiary experiments, participants could report that their motor responses were delayed more for weaker stimuli than for stronger stimuli (second experiment), but the onset timing information was available to them only when they could detect the stimulus (third experiment). This is not paradoxical if one assumes that the brain establishes the time marker representation referring to a time point earlier than detection timing retrospectively or postdictively (i.e., after stimulus detection; Dennett and Kinsbourne, 1992; Eagleman and Sejnowski, 2000; Nishida and Johnston, 2002; Miller and Schwarz, 2006; Shimojo, 2014; Yamada et al., 2015). Since backward referral was proposed by Libet et al. (1979), retrospective processing (postdiction) has been considered a controversial concept by some neuroscientists, since it appears to require very complex neural processing. However, postdiction is a logically reasonable strategy for the brain to accurately estimate event times (Dennett and Kinsbourne, 1992), and it provides a powerful framework to explain a number of counterintuitive perceptual effects, particularly in the context of time perception (Eagleman and Sejnowski, 2000; Nishida and Johnston, 2002; Shimojo, 2014; Yamada et al., 2015). One possible algorithm supporting this is that the brain sets a very low threshold to determine onset timing, but even when the accumulated response exceeds the threshold, this timing information needs to be verified in subsequent processing to be consciously recognized as a real time-marker of the event. The verification is necessary to exclude accidental response increments due to noise. An alternative algorithm is to keep the time course of the accumulated response in memory, and determine the onset timing from the history of response change afterward, perhaps by setting the lowest threshold possible (Miller and Schwarz, 2006). While using the whole response time course for the time marker assignment is computationally optimal (Miller and Schwarz, 2006), it remains controversial whether and how it is actually implemented in the brain.

While our models implicitly assume that the processing after the integrated sensory signals cross a threshold is the same for RT and SJ, we cannot fully exclude possible differences in downstream processing. Namely, even if we assume a similar threshold for SJ and RT tasks (or even a higher threshold for SJ), a smaller scaling factor for the SJ task that compresses the difference in threshold-crossing times might be able to account for the smaller variation in PSS than RT. Although this model seems to be possible at least theoretically, it is not clear whether this kind of compression is implemented in the brain. One issue is whether the compression could be a general mechanism for detecting the timing differences of stimuli. Let us assume a situation where two of the same stimulus type (e.g., two beeps) are presented with a certain interstimulus interval. Given that the two stimuli, and thus the integrated sensory signals, are the same, the difference in timing when the sensory signal evoked by each stimulus crosses a threshold (i.e., time-marker latency) is the same as the interstimulus interval. Therefore, compression of the difference in time-marker latency corresponding to the two stimuli would result in underestimation of the interstimulus interval, which is counterintuitive and does not seem to happen in most cases. Although it might be possible to generate a complex model in which compression occurs in specific situations, such a model might be needlessly complicated. It should also be noted that the compression model needs the postdictive processing that we propose because the difference in time-marker latencies is compressed after the time marker corresponding to each stimulus is detected. Regardless, our integrator model with a relatively lower threshold can account for PSS variation, but does not completely exclude other models; thus, it would be interesting to test other models, including the time compression model, in the future.

In contrast to single-unit electrophysiology, MEG measures population activity and cannot separate neurons sensitive to different motion directions. The integrator in the brain may use neuronal activity for each motion direction rather than the population activity to generate perceptual decisions and judgments of simultaneity. Additionally, we extracted the visual response using the SSP method, but it is possible that the response does not purely reflect the hMT+ time course, but includes the activity of other higher areas. These limitations may partially explain why the physiologically implausible full integrator model was the best. In the future, a single-unit study, which would be free from these limitations, would help to test whether the integrator model is appropriate not only for simple reactions, but also for simultaneity judgments. Further, assessing trial-by-trial simultaneity judgments based on trialwise signals is an important future direction.

While hazard rate is typically controlled in the RT literature, controlling hazard rate in simultaneity judgments is very difficult. This is because an auditory stimulus cues coherent motion in negative SOA trials, while the onset of coherent motion is less predictable in positive SOA trials. This difference in predictability between positive and negative SOAs always exists, by the nature of the task. In the current study, the duration of incoherent motion stimuli was uniformly distributed, which can lead to a nonconstant (increasing) hazard rate (Janssen and Shadlen, 2005; Cui et al., 2009). Therefore, it is possible that temporal predictability of the coherent motion originating from the preceding auditory stimulus, as well as from the nonuniform hazard rate, could have increased the variability of the simultaneity judgments. However, the effects would be constant across different motion conditions, because the stimulus time course was identical in each case. Therefore, the difference in predictability of coherent motion onset cannot affect the main conclusion that the time marker is extracted by setting a relatively low threshold for the integrated MEG signal. In the future, it would be interesting to empirically test the relationship between RT/PSS and the duration of incoherent motion (this analysis is impossible for the current data because the duration of incoherent motion was not recorded).

Several fMRI studies have sought the areas in the brain involved in synchrony detection (Dhamala et al., 2007; Noesselt et al., 2007) by examining the blood oxygenation level-dependent contrast between synchronous and asynchronous multimodal stimuli. Such studies found that a network including the superior temporal sulcus (STS) as well as the sensory areas is involved in synchrony detection. These studies suggest that the time markers extracted from the integrated sensory signals are compared in the STS.

The current study suggests that the time marker for the perception of event timing is assigned when an integrated sensory signal crosses a relatively low threshold, and event timing is retrospectively perceived after stimulus detection. This time marker is considerably independent of the stimulus amplitude, and thus, contributes to accurate timing perception. It may be that the current study does not present direct evidence for an integrated signal in the brain because we measured the signal used as an input to the integrator, rather than the integrated signal itself. Our conclusions are based on the assumption that the integration mechanism exists in the brain, and that its input can be measured by MEG. Discovering an integrated signal used for stimulus detection and for simultaneity judgments would be an important finding that remains to be explored in future research.

## Synthesis

The decision was a result of the Reviewing Editor Kathryn Jeffery and the peer reviewers coming together and discussing their recommendations until a consensus was reached. A fact-based synthesis statement explaining their decision and outlining what is needed to prepare a revision is listed below. The following reviewer(s) agreed to reveal their identity: Erik Cook

This study addresses the link between sensory representations in human visual cortex and the subjective perception of simultaneity. The approach was to model behavioral performance in either simultaneity judgment (SJ) or reaction time detection (RT) tasks using a random-dot motion stimulus. Neural responses to the stimulus were recorded with MEG. The goal was to understand how visual representations are used to determine the temporal order between two stimuli (onset of the random dot motion and a brief auditory stimulus).

The authors find that a simple threshold applied to a filtered version of the MEG sensory representations is predictive of the behavioral performance in both the SJ and RT tasks. Central to their conclusion is that the model's threshold is lower for the PSS compared to the RT task. The model is similar to previous work to account for RT from MEG data (eg Amon 2006), but the application to SJ is novel.

This paper has been previously reviewed and it appears the authors have made good efforts to improve the manuscript. But I still have several concerns about the overall conclusions and the lack of clarity in some portions of the paper.

1) I think the main conclusion that a "time marker for timing perception" occurs prior to "stimulus detection" is a little overstated. As the authors mention on Line 376, because the variance in PSS is smaller than the variance in RT, one would expect the threshold to be lower for PSS versus RT when applied to same sensory responses.

My concern is that this conclusion is built on the major assumption that what happens after the sensory signals cross a threshold is the same for RT and PSS tasks. It would not take much to make the model use approximately the same threshold-crossing to predict both PSS and RT. For example, one could add to the model a simple scaling (ie non-unity slope line) that compressed threshold-crossing times during the PSS task. As is, the model is already adding an offset to the threshold-crossing time, so including a scaling (1 more parameter) is not a big addition.

In addition, the results of the third experiment (which I thought was nicely designed) supports a more parsimonious model that uses the same threshold in both tasks.

My main point is that it is not unreasonable to think that the estimation of SJ may engage different downstream processing of the sensory signals compared to simple detection. Thus, the authors conclusion is somewhat simplistic. I think a more honest assessment of the model results (last sentence in abstract and elsewhere) would be something "Our findings suggest that the brain assigns the time marker for timing perception that is less than or equal to the time of stimulus detection." I think we can safely conclude that thresholds are probably not greater for SJ compared to detection.

Backing off a bit on the literal interpretation of the model results does not reduce the main contribution of the study that PSS can be derived from sensory responses using a model framework (ie filter and threshold of sensory representations) that is also used to predict RT.

2) Clarity of what the model contains would be improved if a block description was included at the top of Fig 3. Something like:

MEG -> 1SD Noise threshold -> Optional integration -> Threshold or peak detection -> Added temporal offset -> RT or PSS.

As is , one has to flip back to the methods to learn the model details, which are important for understanding the basis for the conclusions.

3) Line 203 suggests that some conditions were not used if they did not exceed noise levels. Was the RSS subsequently adjusted to account for the reduced number of conditions? And why not report an average RSS (across conditions) in Figure 3? Although this would not change anything, it would provide the reader an easier to understand error measure (ie the average millisecond error of the model)?

4) When presenting the model in Fig 3A, please remind the reader that the MEG signals were collected only during the SJ task and the RT task was performed outside the MEG.

5) I didn't quite understand the bottom right panel in Figure 2. On Panel D (I think), please indicate the M100 &amp; M200 peaks.

6) Second experiment. Line 424 it is stated that the SJ task was also performed in the second experiment. But the text describing the results of the second experiment largely ignored the PSS results and what they mean. Better clarity is warranted here.

7) In the RT task with the lowest emotion coherence, how do the "missed" trial MEG responses compare to those used in the model? The prediction is that they should be smaller than those associated with a correct detection.

8) I was disappointed to see that after revisions, this manuscript still contained many grammatical errors and several important factual errors. This paper needs to be proofed carefully by the authors. For example, Figure 2 has no panel labels (A, B, C...).

Line 63: ... assigned near the stimulus...

Line 195: The RSS... Is this sentence correct?

Line 375: Is the reference to Figure 4A correct?

Line 508: Should the reference be to Figure 4B?

Line 453: ...responses "until" the accumulated...

Line 475: ...RT task was lower than that for the "direction" task...

Also, many figures contain a mix of example and population results. It would be easier on the reader (ie reviewer) if they labeled panels as "Example Subject" or "Population (N = XX)".
